# Precise anatomical delineation in ultrasound imaging using echo‐spectral interactive networks

**DOI:** 10.1002/acm2.70891

**Published:** 2026-11-08

**Authors:** Yi Xin, Shiyou Wei, Qianping He, Qingqing Ling, Wei Zhu, Wei Jiao, Dandan Ling

**Affiliations:** ^1^ Department of Anesthesiology Fudan University Shanghai Cancer Center Shanghai China; ^2^ Department of Anesthesiology Shanghai Pulmonary Hospital School of Medicine Tongji University Shanghai China; ^3^ OUTCOMES RESEARCH®Consortium Houston Texas USA; ^4^ College of Mathematics and Systems Science Xinjiang University Urumqi China; ^5^ Department of Radiology Shanghai Pulmonary Hospital School of Medicine Tongji University Shanghai China

**Keywords:** medical image analysis, self‐attention, spatial‐frequency fusion, ultrasound image segmentation

## Abstract

**Background:**

Accurate ultrasound (US) segmentation is challenging because speckle noise, low contrast, and ambiguous tissue interfaces often obscure anatomical boundaries. Many conventional CNN‐ and Transformer‐based models operate primarily in the spatial domain and may not explicitly incorporate boundary‐related spectral cues.

**Purpose:**

To develop a spatial–frequency interactive segmentation framework designed to support boundary delineation in US images.

**Methods:**

We introduce the Echo‐Spectral Interactive Transformer (ESI‐Former) block, which uses a Spectrum‐Directed Self‐Attention (SDSA) module to inject differentiable DCT‐derived spectral representations into self‐attention. We further introduce a Skip‐Aware Attention Fusion Module (SAFM) to adaptively fuse encoder skip features with decoder representations. Based on these components, we construct a U‐shaped segmentation architecture termed SF‐EchoNet.

**Results:**

SF‐EchoNet was evaluated on the TN3K thyroid nodule segmentation dataset and the benign subset of the Breast Ultrasound Images (BUSI) dataset. It achieved run‐level mean mIoU scores of 88.01% and 79.58%, with numerically lower run‐level mean HD95 values of 41.31 and 47.22, respectively, than the compared methods. Efficiency evaluation showed 12.24M parameters, 9.98 ms latency, and approximately 100 FPS on an NVIDIA RTX A6000 GPU.

**Conclusions:**

On the two evaluated public datasets, SF‐EchoNet achieved higher run‐level mean overlap scores and numerically lower run‐level mean HD95 values than the compared methods. Paired analyses supported the overlap differences on both datasets, whereas the magnitude of the mean HD95 difference on the BUSI benign subset remained uncertain. Generalizability to other datasets, acquisition settings, and downstream clinical tasks requires further validation.

## 1 INTRODUCTION

Ultrasound (US) imaging represents a ubiquitous modality in contemporary medical diagnostics, favored for its non‐ionizing nature, real‐time feedback, and broad accessibility. It is routinely deployed for the anatomical evaluation of soft tissues, including the thyroid, breast, liver, and cardiovascular systems. Accurate segmentation of anatomical or lesion regions is an important prerequisite for quantitative image analysis, such as size measurement, boundary assessment, and longitudinal comparison. In thyroid nodule and breast lesion ultrasound, reliable contour extraction can provide useful structural information for subsequent clinical interpretation. However, developing robust automated segmentation algorithms for US imaging remains challenging due to the underlying physics of acoustic wave propagation. Sonographic scans are intrinsically affected by speckle interference—a result of sub‐resolution tissue scattering—as well as acoustic shadowing, heterogeneous tissue textures, and diminished edge contrast.^7^ As illustrated in Figure 1, these phenomena can reduce effective contrast and create visually ambiguous interfaces between adjacent structures, complicating the delineation process.

**FIGURE 1:**
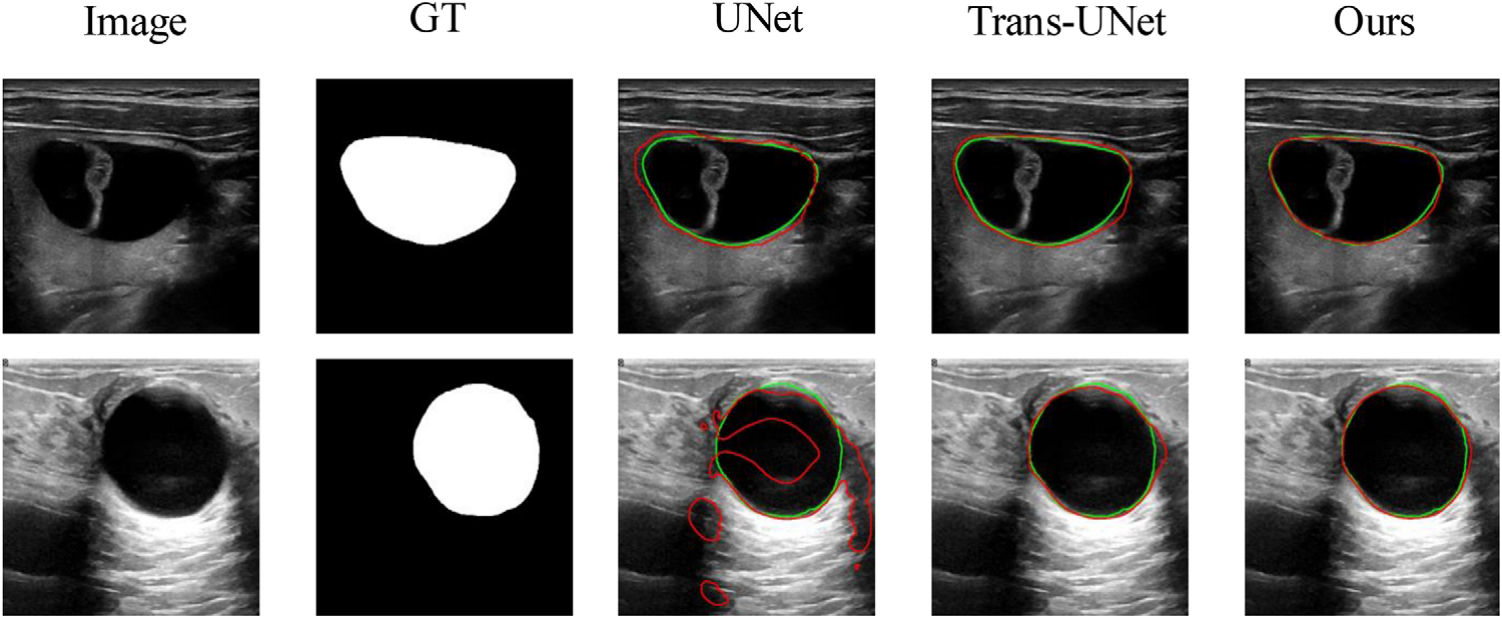
Visual comparison of challenging sonographic scenarios and the corresponding segmentation masks generated by UNet, TransUNet, and our proposed SF‐EchoNet. The ground truth annotations are delineated in green, whereas the algorithmic predictions are outlined in red.

The advent of deep learning has substantially influenced medical image segmentation. Traditional Convolutional Neural Networks (CNNs), particularly UNet,^10^ established strong performance baselines by leveraging an encoder‐decoder topology paired with skip connections to bridge semantic and fine‐grained spatial representations. Subsequent CNN‐centric architectures, such as Attention‐UNet^11^ and UNet++,^12^ have further refined this paradigm through enhanced feature aggregation and localized attention routing. Nevertheless, the locality of convolutional kernels can limit the direct modeling of long‐range spatial dependencies relevant to diffuse anatomical boundaries and large morphological variations in US scans. To address this limitation, the Transformer paradigm^13^ was adapted for vision tasks. Vision Transformers (ViTs)^14^ and hierarchical variants such as the Swin Transformer^15^ use self‐attention to model broader contextual relationships. Hybrid architectures such as TransUNet^16^ and TransFuse^17^ were subsequently developed to combine convolutional feature extraction with Transformer‐based context modeling. Related CNN–Transformer architectures and skip‐connection designs have also been explored for segmentation tasks in other domains.^18^, ^19^ Despite strong performance across several modalities, CNN‐ and Transformer‐based models can still exhibit limitations on challenging US images, particularly in preserving faint edge transitions under pronounced speckle interference. In the selected examples shown in Figure 1, UNet^10^ and TransUNet^16^ produce fragmented or smoothed contours, illustrating remaining challenges in representing weak lesion boundaries.

Frequency‐domain analysis can complement spatial‐domain representations by describing global intensity variation and local transitions. Spectral features may capture periodic texture patterns and intensity gradients that coincide with tissue margins, although these patterns are not specific to anatomical boundaries. Recent deep learning frameworks incorporate frequency information through auxiliary branches, frequency filtering, feature decomposition, or spatial–frequency fusion.^20^, ^21^, ^22^ In these cited designs, frequency descriptors are generally introduced as additional representations rather than used to directly modulate self‐attention in the manner proposed here.

To address these architectural limitations, we propose the Echo‐Spectral Interactive Transformer (ESI‐Former) block, which facilitates interaction between spatial representations and frequency‐domain priors during feature extraction. In contrast to designs that use frequency information only as an auxiliary descriptor, ESI‐Former incorporates spectral cues directly into the attention computation through a **Spectrum‐Directed Self‐Attention (SDSA)** module. By modulating attention weights with differentiable spectral representations, SDSA is designed to emphasize boundary‐related cues. We further introduce a **Skip‐Aware Attention Fusion Module (SAFM)**, which uses joint channel‐wise and spatial attention to integrate encoder skip features with decoder representations with the aim of retaining fine spatial detail and semantic information. By combining SDSA and SAFM within a hierarchical UNet‐like framework, we construct the end‐to‐end **Spatial‐Frequency Echo Network (SF‐EchoNet)** for lesion segmentation in challenging ultrasound images.

In summary, the primary contributions of this work are threefold:

1) We propose the **SDSA** module that embeds frequency‐domain priors directly into self‐attention computation. This design uses spectral cues to modulate spatial attention toward boundary‐related regions.
2) We introduce a **SAFM** to adaptively integrate encoder skip features and decoder representations. By jointly modeling channel‐wise and spatial attentions, SAFM is designed to support the retention of fine structural details during feature reconstruction.
3) We develop the **Spatial‐Frequency Echo Network (SF‐EchoNet)**, which integrates SDSA and SAFM within a UNet‐like architecture. The resulting framework combines explicit spatial–frequency modeling with a relatively compact architecture for ultrasound lesion segmentation.

## 2 PROPOSED METHOD

### 2.1 Overview of SF‐EchoNet

As illustrated in Figure 2, the proposed SF‐EchoNet follows a canonical encoder–decoder architecture tailored for ultrasound image analysis. To extract hierarchical sonographic representations, we adopt the widely used ResNet‐18 as the encoder backbone. The backbone consists of four progressive stages, producing feature maps with downsampling ratios of 4×, 8×, 16×, and 32×, and channel dimensions of 64, 128, 256, and 512, respectively. Such a hierarchical feature extraction strategy enables the network to gradually compress spatial redundancy while capturing increasingly abstract semantic representations, spanning from fine‐grained tissue textures to high‐level anatomical structures.

**FIGURE 2:**
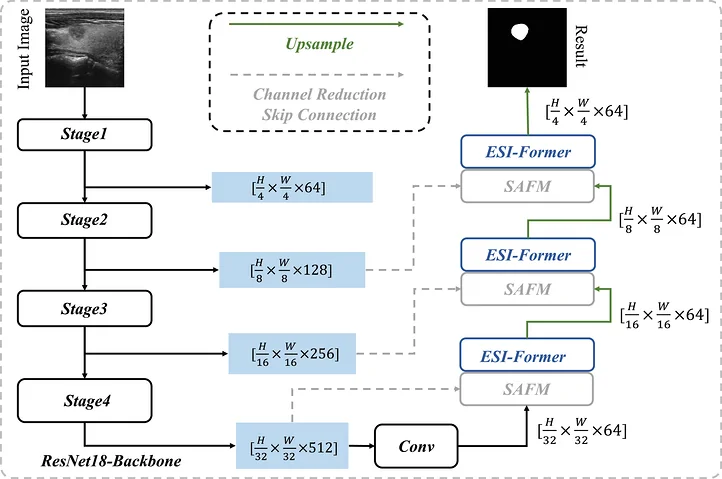
The overall architecture of the proposed SF‐EchoNet.

During the decoding stage, to maintain an efficient computational footprint, the channel dimension of decoder features is unified to 64. Specifically, the multi‐scale encoder features transmitted via skip connections are first projected to 64 channels using a 1×1 bottleneck convolution. The projected encoder features are then fused with the upsampled decoder features through the proposed **SAFM**. By jointly modeling channel‐wise and spatial attentions, SAFM combines encoder skip features and decoder representations with the aim of retaining fine spatial detail and semantic information. On top of the fused features, the **Echo‐Spectral Interactive Transformer (ESI‐Former)** block further processes the feature representations. Within this block, the proposed SDSA module uses frequency‐domain priors to modulate the attention computation toward boundary‐related regions, providing frequency‐guided refinement during feature reconstruction. Finally, the high‐resolution feature maps produced by the last decoder stage are upsampled by a factor of four and passed through a 1×1 convolutional classifier to generate the final lesion probability map. Following Figure 2, the subsequent subsections describe four core components: spectral tokenization, spectrum‐directed self‐attention, multi‐receptive feature enhancement, and skip‐aware decoder fusion.

### 2.2 Spectral tokenization for frequency‐domain prior extraction

#### 2.2.1 DCT‐based spectral tokenization

The spatial feature map is transformed into a compact frequency‐domain representation by computing a channel‐mean feature map and applying a differentiable DCT. These spectral tokens provide lightweight global frequency guidance without replacing the original spatial features. Given an intermediate feature tensor $X\in R^{B\times C\times H\times W}$, we first reshape the spatial feature map into a token sequence $X_{t}\in R^{B\times N\times C}$, where N=H×W denotes the number of spatial tokens. To obtain a compact spectral representation while reducing computational overhead, we compute the channel‐wise mean activation of the token sequence, resulting in

(1) $$X_{\text{mean}}=\text{Mean}\left(X_{t},\text{dim}=C\right),\;X_{\text{mean}}\in R^{B\times N}.$$

The mean sequence is zero‐padded, when necessary, to the nearest square length and reshaped into a two‐dimensional grid. We then implement a differentiable Type‐II 2D DCT using FFT operations, retain the first N flattened coefficients, and clip the resulting values to [−10,10]. Finally, L2 normalization with a small numerical‐stability constant produces $X_{\text{out}}\in R^{B\times N}$. This normalized spectral vector preserves the token layout and serves as the frequency prior for subsequent attention modulation in SDSA. Because the original spatial stream is preserved, the channel‐averaged spectral branch supplies auxiliary guidance without replacing channel‐specific semantic representations.

## 3 Spectrum‐directed self‐attention for spatial‐frequency interaction

Figure 3 illustrates the internal structure of the proposed Echo‐Spectral Interactive Transformer (ESI‐Former) block. Following the design philosophy of modern vision Transformers,^13^, ^14^ the ESI‐Former block consists of two primary computational sub‐units: a SDSA module and a Multi‐Receptive Feed‐Forward Network (MR‐FFN) block. Each sub‐unit is encapsulated with Batch Normalization and residual connections.^23^ Given an input acoustic feature tensor $X\in R^{B\times C\times H\times W}$, the SDSA output is added to X through a standard BatchNorm–residual wrapper. The SDSA module introduces frequency‐domain priors into the attention computation so that spatial correlations are conditioned on spectral information while global contextual modeling is retained. The detailed computational flow of SDSA is illustrated in Figure 4.

**FIGURE 3:**
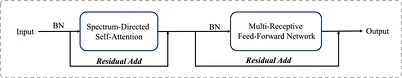
Internal schematic of the proposed ESI‐Former block.

**FIGURE 4:**
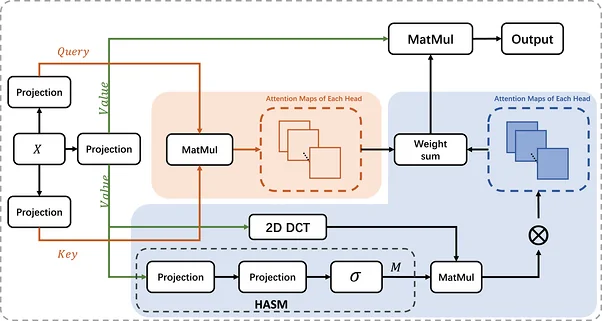
Schematic of the SDSA module.

### 3.1 Spectrum‐directed self‐attention

Conventional Multi‐Head Self‐Attention (MHSA)^13^ constructs correlation matrices from spatial feature similarities alone and may therefore be affected by speckle‐related feature ambiguity in ultrasound images. SDSA injects spectral tokens into the self‐attention computation so that spatial correlations are modulated by frequency‐domain information while the global contextual modeling ability of Transformer attention is retained.

The input tensor is first projected into query, key, and value representations. Following the window‐based strategy of the Swin Transformer,^15^ these representations are partitioned into non‐overlapping windows and arranged into h attention heads. Each window contains $N_{w}=\text{window}\_{\text{size}}^{2}$ tokens, and each head has dimension d=C/h.

Within each window, the conventional spatial attention matrix is computed as

(2) $$A_{\text{sp}}=\frac{Q_{w}K_{w}^{⊤}}{\sqrt{d}}.$$

To incorporate frequency priors, the windowed value tensor $V_{w}$ is transformed by the differentiable DCT extractor. The obtained spectral representation $X_{\text{freq}}$ is subsequently processed by a **Head‐Adaptive Spectral Modulator (HASM)**, which predicts head‐specific modulation weights:

(3) $$M=\sigma (\text{Linear}\left(\phi \left(\text{Linear}\left(X_{\text{freq}}\right)\right)\right)).$$

These spectral modulation weights are used to generate the frequency attention component

(4) $$A_{\text{freq}}=M⊙A_{\text{sp}},$$

where ⊙ denotes element‐wise multiplication.

To adaptively balance spatial and spectral cues, we introduce a learnable gating parameter α:

(5) $$A=\left(1-\sigma \left(\alpha \right)\right)A_{\text{sp}}+\sigma \left(\alpha \right)A_{\text{freq}}.$$

The resulting attention matrix is applied to $V_{w}$, after which the windowed outputs are reassembled into a global feature tensor with the same spatial and channel dimensions as the input.

Through this formulation, the spectral branch functions as an attention‐guidance signal and provides a mechanism for incorporating frequency‐domain evidence into spatial correlations.

To alleviate the loss of global interactions caused by window partitioning, we further introduce an adaptive cross‐window propagation mechanism (Figure 5). Specifically, we perform 1D average pooling along the horizontal and vertical directions to propagate contextual information across neighboring windows. The responses from the two orthogonal directions are combined with two learnable scaling parameters, enabling cross‐window context propagation through low‐complexity pooling operations.

**FIGURE 5:**
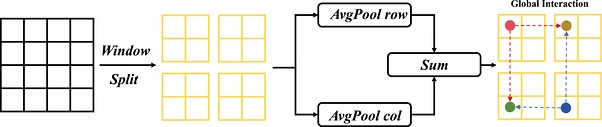
Illustration of the adaptive cross‐window propagation mechanism.

## 4 Multi‐receptive feed‐forward network

### 4.1 MR‐FFN

Following the attention operation, the intermediate feature $X^{\prime }$ is refined by MR‐FFN within a standard BatchNorm–residual wrapper. The resulting tensor $Y\in R^{B\times C\times H\times W}$ is the final output of the ESI‐Former block. By aggregating local patterns at different receptive‐field scales, MR‐FFN complements spectral‐guided global attention and reinforces local texture transitions and fine boundary details.

Different from the conventional point‐wise feed‐forward network used in standard Transformers,^13^ MR‐FFN processes $X_{in}\in R^{B\times C\times H\times W}$ through three parallel depth‐wise convolutional branches with 3×3, 5×5, and 7×7 kernels. Their outputs are aggregated as

(6) $$X_{add}=\sum_{k\in \{3,5,7\}}\mathrm{DW}-{\mathrm{Conv}}_{k\times k}\left(X_{in}\right).$$

The aggregated feature is then passed through two 1×1 convolutions: the first expands the channel dimension to 4C, and the second projects it back to C, yielding a locally refined multi‐receptive representation. In this way, the ESI‐Former block combines frequency‐guided global dependencies with multi‐scale local context for ultrasound boundary delineation.

## 5 Skip‐aware attention fusion for decoder reconstruction

To integrate multi‐scale representations within the encoder–decoder architecture, we introduce a SAFM. This module is designed to adaptively fuse encoder features transmitted via skip connections with the corresponding decoder features, allowing the network to combine fine‐grained spatial details with decoder semantic representations during feature reconstruction.

As illustrated in Figure 6, let the encoder skip feature and the decoder feature be denoted as $X_{enc}$ and $X_{dec}$, respectively, where both tensors belong to $R^{B\times C\times H\times W}$. These two feature maps are first concatenated along the channel dimension and projected through a 1×1 convolution to produce an initial fused representation X:

(7) $$X={\mathrm{Conv}}_{1\times 1}\;\left([X_{enc}\;∥\;X_{dec}]\right).$$

**FIGURE 6:**
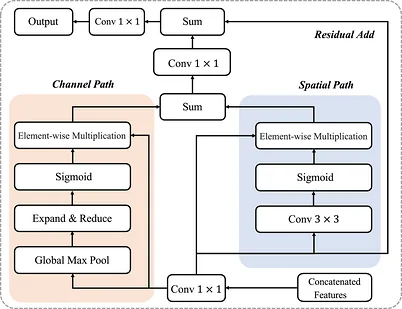
Architecture of the SAFM.

To refine the fused representation, the composite tensor X is processed through two parallel attention pathways: a *Channel Path* and a *Spatial Path*.

Within the **Channel Path**, Global Max Pooling (GMP) is applied to extract salient channel responses, yielding a compact descriptor $z\in R^{B\times C\times 1\times 1}$. A bottleneck transformation consisting of expansion and reduction fully connected layers, connected by a ReLU activation δ, is then employed to estimate the channel attention weights $s_{c}$:

(8) $$s_{c}=\sigma \left({\mathrm{FC}}_{reduce}\left(\delta \left({\mathrm{FC}}_{expand}\left(z\right)\right)\right)\right).$$

The channel weights recalibrate the fused representation by element‐wise multiplication, producing $X_{\mathrm{ch}}=X⊙s_{c}$.

In parallel, the **Spatial Path** models spatial dependencies using a 3×3 convolutional layer to generate a spatial attention mask $s_{s}$:

(9) $$s_{s}=\sigma \left({\mathrm{Conv}}_{3\times 3}\left(X\right)\right),$$

which is applied element‐wise to emphasize spatially informative regions, producing $X_{\mathrm{sp}}=X⊙s_{s}$.

Finally, the channel‐refined and spatial‐refined features are aggregated. Specifically, $X_{\mathrm{ch}}$ and $X_{\mathrm{sp}}$ are summed, compressed by a 1×1 convolution, and combined with the initial fused feature X through a residual connection. The final output Y is computed as:

(10) $$Y={\mathrm{Conv}}_{1\times 1}\left({\mathrm{Conv}}_{1\times 1}\left(X_{\mathrm{ch}}+X_{\mathrm{sp}}\right)+X\right).$$

By jointly modeling channel‐wise and spatial attentions, SAFM is designed to integrate encoder skip features and decoder representations while retaining fine structural details and semantic information. Within SF‐EchoNet, this skip‐aware decoding stage connects spatial encoding, spectral tokenization, spectrum‐guided attention, and local feature enhancement in a unified segmentation framework.

## 6 RESULTS

### 6.1 Experiment settings

#### 6.1.1 Dataset

In this section, we evaluate our framework on two publicly available ultrasound image datasets: the TN3K thyroid nodule segmentation dataset and the benign subset of the Breast Ultrasound Images (BUSI) dataset. (1) **TN3K Thyroid Nodule Segmentation Dataset**: TN3K comprises 3,493 thyroid ultrasound images with corresponding nodule segmentation masks.^8^ The official release provides 2,879 development (training/validation) images and 614 held‐out test images. Accurate thyroid nodule segmentation can support quantitative image assessment, including measurements of lesion size, shape, and boundary characteristics. (2) **Breast Ultrasound Images (BUSI) Dataset—Benign Subset**: The complete BUSI dataset contains normal, benign, and malignant breast ultrasound images.^9^ This study used only the 437 benign images with pixel‐level lesion annotations. These images were randomly partitioned at the image level into 305 training and 132 testing images at a 7:3 ratio.

#### 6.1.2 Evaluation metrics

To quantify segmentation performance, we used six standard metrics: Mean Intersection over Union (mIoU), Dice Similarity Coefficient (DSC), Accuracy (Acc), Sensitivity (Sen), Specificity (Spe), and the 95th‐percentile Hausdorff Distance (HD95).^24^ The first five metrics were computed from the pixel‐level true‐positive, true‐negative, false‐positive, and false‐negative counts using their conventional definitions; HD95 was used to assess boundary localization fidelity. Unless otherwise stated, values reported as mean ± standard deviation summarize five independent training runs with distinct random seeds.

To assess the statistical reliability of the reported improvements, image‐level paired analyses were performed between SF‐EchoNet and the strongest competing method, BDFormer. For each test image, mIoU, mDSC, and HD95 were calculated for both methods. Since image‐level segmentation metrics may not follow a normal distribution, paired Wilcoxon signed‐rank tests were used for statistical significance testing. In addition, non‐parametric bootstrap resampling with 5000 image‐level replicates was conducted to estimate 95% confidence intervals (CIs) for both the mean metric values and the paired differences. Holm–Bonferroni correction was applied across the tested metrics within each dataset. For mIoU and mDSC, the paired difference was defined as SF‐EchoNet minus BDFormer; for HD95, the paired difference was defined as BDFormer minus SF‐EchoNet, so that a positive value consistently favors SF‐EchoNet. The run‐level summary values in Table 1 and the image‐level statistics in Table 2 use different aggregation units and therefore are not numerically interchangeable; differences can be substantial, particularly for HD95.

**TABLE 1:** Comparative experimental results on the TN3K thyroid nodule segmentation dataset (2879 development (training/validation) images; 614 held‐out test images) and the BUSI benign subset (437 images; 305 training / 132 testing).

Dataset	Method	mIoU↑	DSC↑	Acc↑	Spe↑	Sen↑	HD95↓
TN3K	${\mathrm{UNet}}_{\left(2015\right)}$ ^10^	77.02 ± 0.34	86.03 ± 0.27	94.11 ± 0.18	96.72 ± 0.22	74.98 ± 0.53	90.71 ± 2.10
	UNet++ 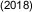 ^12^	77.56 ± 0.46	86.44 ± 0.33	94.19 ± 0.21	96.54 ± 0.25	77.04 ± 0.48	91.11 ± 1.84
	${\mathrm{SANet}}_{\left(2021\right)}$ ^25^	84.82 ± 0.41	91.39 ± 0.36	96.41 ± 0.23	98.25 ± 0.19	83.01 ± 0.44	48.57 ± 1.05
	TransFuse_(2021)_ ^17^	81.93 ± 0.37	89.45 ± 0.31	95.92 ± 0.19	**99.09** ± **0.14**	72.84 ± 0.59	53.91 ± 1.46
	Swin‐UNet_(2022)_ ^26^	78.64 ± 0.42	86.91 ± 0.35	94.53 ± 0.24	97.02 ± 0.26	75.42 ± 0.57	84.19 ± 2.14
	Focal‐UNETR_(2023)_ ^27^	79.83 ± 0.29	88.02 ± 0.41	95.13 ± 0.22	97.81 ± 0.24	75.46 ± 0.48	72.64 ± 1.93
	Trans‐UNet_(2024)_ ^16^	80.51 ± 0.33	88.49 ± 0.29	95.35 ± 0.18	98.12 ± 0.21	75.16 ± 0.55	64.13 ± 1.67
	I2U‐Net_(2024)_ ^28^	78.91 ± 0.40	87.02 ± 0.35	94.71 ± 0.23	97.15 ± 0.25	75.78 ± 0.49	82.33 ± 2.21
	CSCA‐UNet_(2024)_ ^29^	79.35 ± 0.39	87.45 ± 0.31	94.88 ± 0.21	97.27 ± 0.23	76.11 ± 0.45	80.84 ± 1.92
	MALUNet_(2022)_ ^30^	74.41 ± 0.47	84.01 ± 0.44	93.52 ± 0.26	97.03 ± 0.29	67.93 ± 0.66	75.63 ± 2.37
	DSU‐Net_(2025)_ ^31^	81.29 ± 0.32	89.04 ± 0.30	95.51 ± 0.20	97.99 ± 0.22	77.44 ± 0.46	69.44 ± 1.58
	MSCB‐UNet_(2025)_ ^32^	79.72 ± 0.35	87.63 ± 0.28	95.01 ± 0.19	97.31 ± 0.21	76.53 ± 0.42	78.41 ± 1.89
	BDFormer_(2025)_ ^33^	85.08 ± 0.31	91.56 ± 0.27	96.47 ± 0.16	98.23 ± 0.20	83.64 ± 0.38	51.98 ± 1.18
	SF‐${\mathrm{EchoNet}}_{\left(Ours\right)}$	**88.01** ± **0.25**	**93.38** ± **0.22**	**97.25** ± **0.14**	98.79 ± 0.18	**86.08** ± **0.33**	**41.31** ± **0.97**
BUSI‐benign	UNet_(2015)_ ^10^	63.83 ± 0.42	73.57 ± 0.38	94.38 ± 0.16	98.61 ± 0.12	39.46 ± 0.71	133.72 ± 1.25
	UNet++ 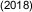 ^12^	64.11 ± 0.37	73.86 ± 0.30	94.45 ± 0.14	98.66 ± 0.10	39.82 ± 0.74	126.01 ± 1.32
	${\mathrm{SANet}}_{\left(2021\right)}$ ^25^	69.42 ± 0.35	79.15 ± 0.29	95.36 ± 0.12	98.85 ± 0.09	50.16 ± 0.63	71.74 ± 1.08
	TransFuse_(2021)_ ^17^	70.41 ± 0.28	80.04 ± 0.33	95.64 ± 0.10	**99.12** ± **0.08**	50.53 ± 0.56	64.97 ± 1.04
	Swin‐UNet_(2022)_ ^26^	64.35 ± 0.45	74.01 ± 0.41	94.52 ± 0.17	98.63 ± 0.13	40.11 ± 0.68	124.38 ± 1.41
	Focal‐UNETR_(2023)_ ^27^	68.81 ± 0.31	78.64 ± 0.27	95.04 ± 0.12	98.38 ± 0.11	51.78 ± 0.59	97.05 ± 1.16
	Trans‐UNet_(2024)_ ^16^	75.23 ± 0.26	84.22 ± 0.24	96.28 ± 0.11	98.94 ± 0.07	61.86 ± 0.49	69.72 ± 0.98
	I2U‐Net_(2024)_ ^28^	68.05 ± 0.33	77.92 ± 0.29	95.12 ± 0.13	98.75 ± 0.08	47.56 ± 0.64	98.67 ± 1.24
	CSCA‐UNet_(2024)_ ^29^	67.72 ± 0.40	77.52 ± 0.36	95.08 ± 0.12	98.79 ± 0.10	46.83 ± 0.68	70.13 ± 1.22
	MALUNet_(2022)_ ^30^	71.41 ± 0.29	81.03 ± 0.27	95.36 ± 0.11	98.17 ± 0.10	58.98 ± 0.52	79.76 ± 0.96
	DSU‐Net_(2025)_ ^31^	67.79 ± 0.38	77.61 ± 0.34	95.09 ± 0.13	98.79 ± 0.09	47.08 ± 0.61	100.51 ± 1.35
	MSCB‐UNet_(2025)_ ^32^	71.24 ± 0.30	80.85 ± 0.28	95.51 ± 0.14	98.56 ± 0.09	55.90 ± 0.57	80.28 ± 1.11
	BDFormer_(2025)_ ^33^	75.72 ± 0.25	84.64 ± 0.23	96.18 ± 0.10	98.48 ± 0.08	66.41 ± 0.47	63.57 ± 0.94
	SF‐${\mathrm{EchoNet}}_{\left(Ours\right)}$	**79.58** ± **0.21**	**87.62** ± **0.18**	**96.75** ± **0.09**	98.36 ± 0.07	**75.88** ± **0.45**	**47.22** ± **0.82**

*Note*: Bold indicates the best performance, and underline indicates the second best.

**TABLE 2:** Image‐level paired statistical comparison between SF‐EchoNet and BDFormer.

Dataset	Metric	SF‐EchoNet mean [95% CI]	BDFormer mean [95% CI]	Paired diff. [95% CI]	Wilcoxon p	Adj. p
TN3K	mIoU	84.55 [83.56, 85.47]	80.38 [79.30, 81.43]	4.17 [3.39, 4.93]	5.22e‐29	1.57e‐28
TN3K	mDSC	89.74 [88.85, 90.60]	86.53 [85.52, 87.48]	3.22 [2.54, 3.89]	6.34e‐27	1.27e‐26
TN3K	HD95	47.54 [41.62, 54.11]	60.53 [54.69, 67.16]	12.99 [7.12, 19.07]	2.75e‐15	2.75e‐15
BUSI‐benign	mIoU	83.03 [80.16, 85.86]	79.82 [76.80, 82.67]	3.21 [1.75, 4.79]	7.60e‐07	2.28e‐06
BUSI‐benign	mDSC	87.52 [84.71, 90.14]	85.23 [82.44, 87.86]	2.29 [0.90, 3.81]	2.69e‐06	5.38e‐06
BUSI‐benign	HD95	85.17 [58.58, 115.70]	86.39 [62.58, 113.71]	1.22 [‐18.78, 19.03]	8.51e‐04	8.51e‐04

*Note*: Confidence intervals were estimated using 5000 image‐level bootstrap replicates. Paired significance was tested using the Wilcoxon signed‐rank test, with Holm–Bonferroni correction across metrics within each dataset. The image‐level statistics reported here and the run‐level summary values in Table 1 use different aggregation units and therefore are not numerically interchangeable, particularly for HD95. For HD95, the paired difference is defined as BDFormer minus SF‐EchoNet, so a positive value favors SF‐EchoNet.

#### 6.1.3 Implementation details

All experiments were executed on an Ubuntu 22.04 workstation equipped with a single NVIDIA RTX A6000 GPU (48GB VRAM). During training, we applied on‐the‐fly data augmentation, including vertical/horizontal flipping and random spatial rotations. The optimization objective was defined as a linear combination of Cross‐Entropy (CE) loss and Dice loss. Network parameters were optimized using the AdamW solver with an initial learning rate of 0.001, which was dynamically decayed following a cosine annealing schedule. The training process spanned 45 epochs with a batch size of 16. Prior to network ingestion, all sonographic inputs were uniformly resized to 512×512 pixels.

For computational efficiency evaluation, all models were benchmarked on an NVIDIA RTX A6000 GPU with an input resolution of 512×512 and a batch size of 1. Each model was first warmed up for 30 iterations, and the average inference latency was then measured over 100 repeated forward passes. Frames per second (FPS) was calculated as the reciprocal of the measured latency. The number of parameters, FLOPs, inference latency, peak GPU memory usage, and FPS were reported to provide a comprehensive assessment of computational efficiency. FLOPs were estimated using fvcore; since FFT/DCT‐related operators may not be fully counted by FLOPs profilers, the measured latency and FPS were treated as the primary indicators of practical inference efficiency.

### 6.2 Comparison with existing methods

Table 1 summarizes the quantitative comparison of segmentation architectures on both datasets. On the TN3K thyroid nodule segmentation dataset (2,879 development (training/validation) images; 614 held‐out test images), SF‐EchoNet achieved the highest primary overlap scores, including an mIoU of 88.01% and a DSC of 93.38%, and reduced HD95 to 41.31. The improvement in boundary agreement is practically relevant because boundary errors can affect image‐derived measurements such as lesion size, shape, and contour irregularity. Nevertheless, the present experiments do not include radiotherapy‐specific datasets, dosimetric endpoints, observer‐variability studies, or downstream clinical task validation. Therefore, these results should be interpreted as evidence of improved segmentation fidelity on public ultrasound benchmarks rather than direct evidence of improved treatment planning or clinical outcomes.

Table 2 reports the image‐level paired statistical comparison between SF‐EchoNet and BDFormer. On TN3K, SF‐EchoNet achieved consistently higher image‐level mIoU and mDSC and lower HD95 than BDFormer. The paired improvements remained statistically significant after Holm–Bonferroni correction, and the bootstrap 95% CIs of the paired differences entirely favored SF‐EchoNet. Specifically, SF‐EchoNet improved mIoU by 4.17 percentage points, mDSC by 3.22 percentage points, and reduced HD95 by 12.99 pixels on average.

On the BUSI benign subset, SF‐EchoNet also achieved significantly higher image‐level mIoU and mDSC than BDFormer, with differences of 3.21 and 2.29 percentage points, respectively. The bootstrap 95% CIs of these overlap‐based differences excluded zero. For HD95, the Wilcoxon signed‐rank test remained significant after correction, suggesting a paired‐rank tendency toward lower boundary error. However, the bootstrap 95% CI of the mean HD95 difference crossed zero, indicating that the magnitude of the boundary‐distance difference on the BUSI benign subset was uncertain because of the smaller test set and the larger variability of HD95. Thus, these results provide stronger evidence for overlap‐based differences than for a consistent reduction in boundary‐distance error.

As shown in Table 3, SF‐EchoNet combines competitive segmentation results with a relatively small computational footprint. The architecture incorporates the SDSA module, the MR‐FFN block, and SAFM while using 12.24M parameters and 12.54G FLOPs. Its parameter count is approximately 12% of those of Trans‐UNet and BDFormer. SF‐EchoNet reached an average latency of 9.98 ms and 100.2 FPS under the tested setting, with higher inference throughput than TransFuse, Trans‐UNet, Focal‐UNETR, BDFormer, and several recent UNet variants in this benchmark. It also required 141.5 MB peak GPU memory, the second‐lowest value in the table after MALUNet. Thus, under the reported hardware and input configuration, SF‐EchoNet achieved the reported mIoU and DSC values alongside comparatively low resource use.

**TABLE 3:** Computational efficiency comparison with representative segmentation models.

Model	Params (M)	FLOPs (G)	Latency (ms)	FPS	GPU Mem (MB)
UNet_(2015)_ ^10^	31.04	192.94	17.17	58.3	663.6
UNet++ 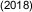 ^12^	9.16	138.63	21.85	45.8	520.1
${\mathrm{SANet}}_{\left(2021\right)}$ ^25^	23.90	24.15	7.63	131.1	309.1
TransFuse_(2021)_ ^17^	144.19	144.06	43.57	23.0	863.6
Swin‐UNet_(2022)_ ^26^	27.17	34.21	15.78	63.4	360.7
Focal‐UNETR_(2023)_ ^27^	108.40	6.65	25.53	39.2	542.1
Trans‐UNet_(2024)_ ^16^	105.91	168.70	32.90	30.4	605.8
I2U‐Net_(2024)_ ^28^	29.65	33.61	10.77	92.9	281.0
CSCA‐UNet_(2024)_ ^29^	35.28	48.27	67.82	14.7	506.0
MALUNet_(2022)_ ^30^	0.18	0.36	6.73	148.5	52.8
DSU‐Net_(2025)_ ^31^	35.69	47.38	22.03	45.4	2315.1
MSCB‐UNet_(2025)_ ^32^	103.56	240.43	259.77	3.8	4329.8
BDFormer_(2025)_ ^33^	103.83	118.80	125.82	7.9	2461.8
**SF‐EchoNet** 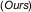	**12.24**	**12.54**	**9.98**	**100.2**	**141.5**

*Note*: All models were evaluated on an NVIDIA RTX A6000 GPU using an input size of 512×512 and batch size of 1. Latency was averaged over 100 repeated forward passes after 30 warm‐up iterations.

Bold values indicate the best performance in each column.

Figure 7 provides qualitative comparisons on representative images from the TN3K thyroid nodule dataset (panel a) and the BUSI benign subset (panel B). In the displayed examples, conventional CNN‐based frameworks (e.g., UNet, UNet++) produce fragmented or jagged masks in some regions with low acoustic contrast, whereas Transformer architectures (e.g., Trans‐UNet, Focal‐UNETR) generate smoother predictions but miss portions of some weak lesion margins. BDFormer produces relatively cohesive boundaries but under‐segments some diffuse targets in these examples. SF‐EchoNet more closely follows the expert contours and produces more coherent breast‐lesion contours in several displayed cases. These observations remain limited to qualitative segmentation assessment on the selected cases.

**FIGURE 7:**
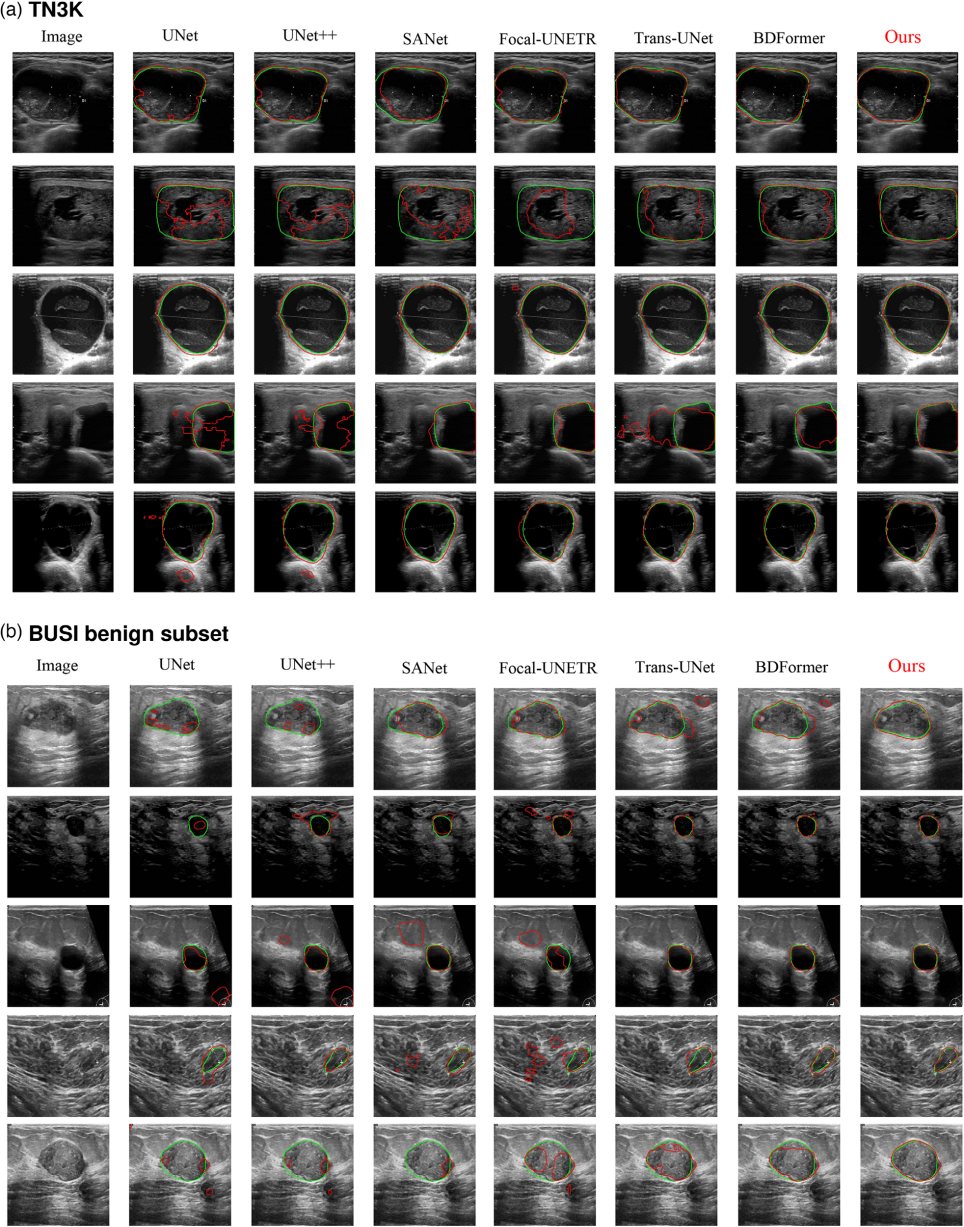
Qualitative segmentation comparisons on (a) TN3K and (b) the BUSI benign subset. Green contours denote ground‐truth masks, and red contours denote model predictions.

On the BUSI benign subset, SF‐EchoNet achieved run‐level mean mIoU and DSC values of 79.58% and 87.62%, respectively, which were higher than those of the compared Transformer‐based methods under the tested setting. The HD95 result suggests a paired‐rank tendency toward reduced boundary deviation, but the bootstrap confidence interval of the paired mean HD95 difference indicates that its magnitude remains uncertain. Compared with BDFormer, SF‐EchoNet also obtained higher sensitivity, corresponding to greater predicted lesion‐region coverage in this experimental setting. These findings are limited to segmentation accuracy and do not establish malignant feature discrimination, radiomic robustness, or diagnostic superiority, since no pathological classification or downstream radiomics analysis was performed.

## 7 DISCUSSION

SF‐EchoNet provides a spatial‐frequency interactive framework for two‐dimensional ultrasound image segmentation. In the run‐level summaries, SF‐EchoNet obtained the highest mIoU and DSC and the lowest mean HD95 among the evaluated methods on the TN3K dataset and the BUSI benign subset. Paired image‐level analyses showed statistically significant differences in mIoU and mDSC on both datasets and in HD95 on TN3K. For HD95 on the BUSI benign subset, the corrected Wilcoxon test was significant, but the bootstrap 95% confidence interval of the mean paired difference crossed zero; therefore, the magnitude of this boundary‐distance difference remains uncertain. The observed frequency‐response patterns were associated with lesion‐boundary regions, but these descriptive analyses do not establish a causal imaging‐physics mechanism or robustness to specific ultrasound degradations. The current experiments evaluate segmentation performance only; whether these results translate into more reliable radiomics, diagnostic classification, treatment planning, or clinical workflow efficiency requires dedicated downstream validation.

### 7.1 Ablation of spectral prior integration

To assess the contribution of frequency‐domain priors, we conducted a targeted ablation study within the ESI‐Former block. As summarized in Table 4, the “No spectral guidance” variant produced lower overlap metrics and higher HD95 values on both datasets. We also evaluated “Late spectral fusion (concat.)” and “Late spectral fusion (addition),” which incorporate frequency features at the aggregation stage. These late spectral‐fusion baselines produced better results than the no‐guidance variant for the reported metrics but remained below the proposed SDSA module under the present experimental setting. Thus, direct spectral integration was associated with better segmentation metrics than the tested alternatives. This ablation does not independently establish that SDSA selectively suppresses speckle noise or identify the mechanism responsible for the observed differences.

**TABLE 4:** Ablation study of spectral‐prior integration within the SDSA module.

Dataset	Method	mIoU↑	DSC↑	Acc↑	Spe↑	Sen↑	HD95↓
TN3K	**SDSA (proposed)**	**88.01** ± **0.25**	**93.38** ± **0.22**	**97.25** ± **0.14**	**98.79** ± **0.18**	**86.08** ± **0.33**	**41.31** ± **0.97**
	Late spectral fusion (concat.)	87.42 ± 0.22	92.97 ± 0.18	97.02 ± 0.09	98.25 ± 0.08	85.66 ± 0.31	42.15 ± 0.98
	Late spectral fusion (addition)	86.95 ± 0.25	92.51 ± 0.19	96.81 ± 0.10	97.93 ± 0.10	85.12 ± 0.34	43.04 ± 1.04
	No spectral guidance	86.11 ± 0.28	91.87 ± 0.23	96.44 ± 0.12	97.41 ± 0.11	84.25 ± 0.36	44.76 ± 1.21
BUSI‐benign	**SDSA (proposed)**	**79.58** ± **0.21**	**87.62** ± **0.18**	**96.75** ± **0.09**	**98.36** ± **0.07**	**75.88** ± **0.45**	**47.22** ± **0.82**
	Late spectral fusion (concat.)	78.94 ± 0.24	86.97 ± 0.21	96.39 ± 0.10	97.85 ± 0.09	75.01 ± 0.47	48.11 ± 1.02
	Late spectral fusion (addition)	78.12 ± 0.27	86.13 ± 0.24	96.08 ± 0.11	97.48 ± 0.10	74.32 ± 0.49	49.07 ± 1.09
	No spectral guidance	77.45 ± 0.29	85.48 ± 0.26	95.74 ± 0.12	97.06 ± 0.11	73.68 ± 0.52	50.16 ± 1.24

*Note*: Bold indicates the best result among the tested configurations.

Figure 8 presents qualitative Grad‐CAM visualizations.^34^ In the displayed examples, the variant without spectral guidance shows more spatially diffuse responses and less coherent predicted contours, whereas the complete model shows responses that are more concentrated near lesion regions. Because these observations are based on selected qualitative examples and Grad‐CAM is not a direct measure of causal feature use, they should be interpreted as supporting visual evidence rather than proof that the spectral pathway distinguishes anatomical boundaries from speckle noise or guarantees performance under severely degraded imaging conditions.

**FIGURE 8:**
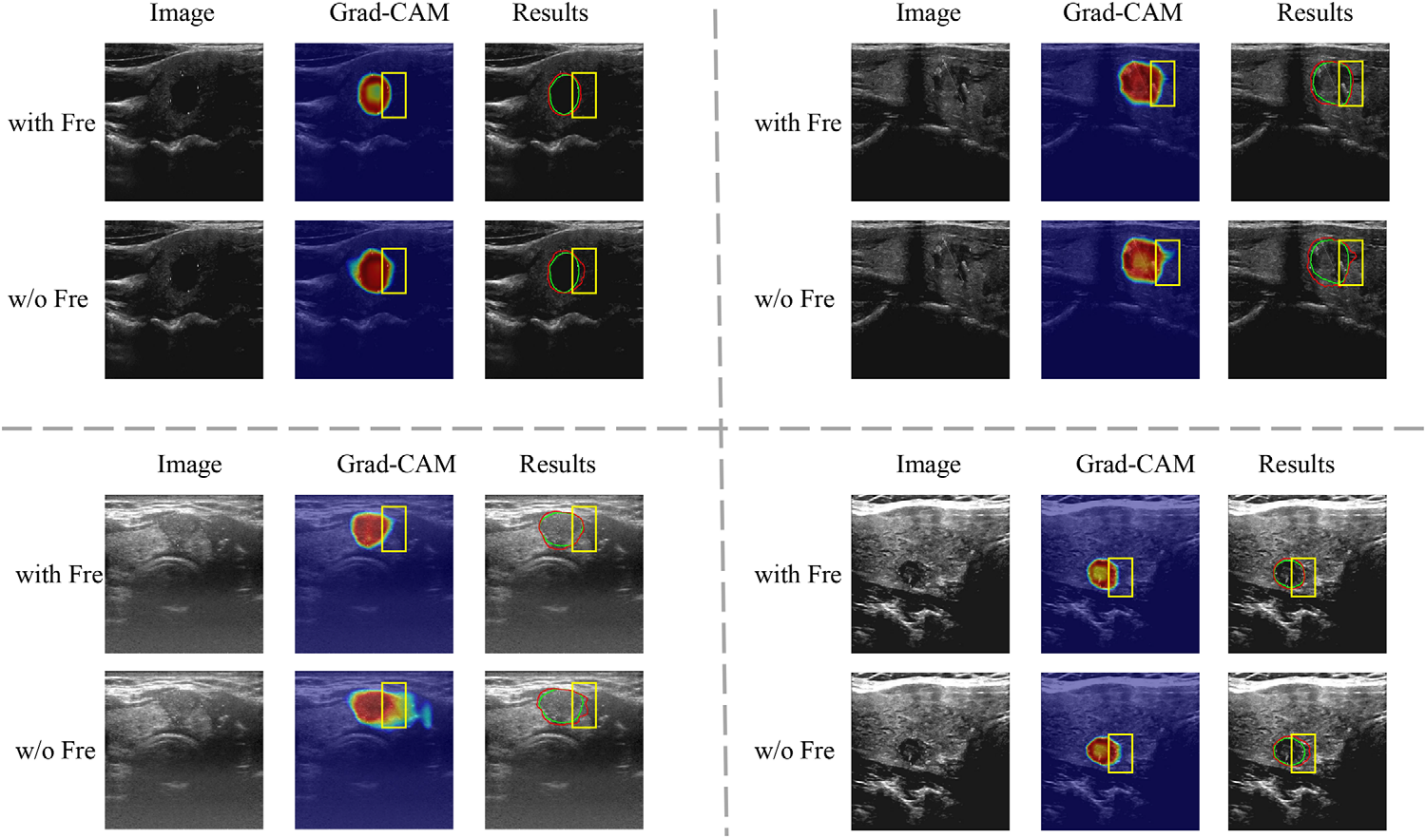
Qualitative ablation of spectral guidance. From left to right: original ultrasound scan, Grad‐CAM attention heatmap, and segmentation output. The embedded labels “w/o Fre” and “with Fre” denote no spectral guidance and the complete SDSA configuration, respectively. Green contours denote ground truth; red contours indicate algorithmic predictions.

### 7.2 Frequency‐band behavior and interpretability

The following analyses characterize associations between the spectral responses produced by SDSA and lesion‐boundary regions. Different radial frequency bands were analyzed in relation to lesion boundaries, and low‐, mid‐, and high‐frequency bands exhibited different spatial response patterns in the analyzed samples. The low‐frequency responses were concentrated in broad image regions, the mid‐frequency responses were more prominent near some local transitions and lesion boundaries, and the high‐frequency responses contained finer and more spatially scattered patterns. These descriptive observations do not assign a unique functional role to any frequency band.

As shown in Figure 9, the displayed low‐frequency responses are spatially broad, whereas the mid‐frequency responses are stronger around some lesion boundaries and local tissue transitions. The high‐frequency responses are finer but also more spatially scattered. The displayed SDSA response is not uniformly distributed and is more prominent near some boundary‐related regions; this descriptive observation does not establish selective or causal use of frequency information.

**FIGURE 9:**
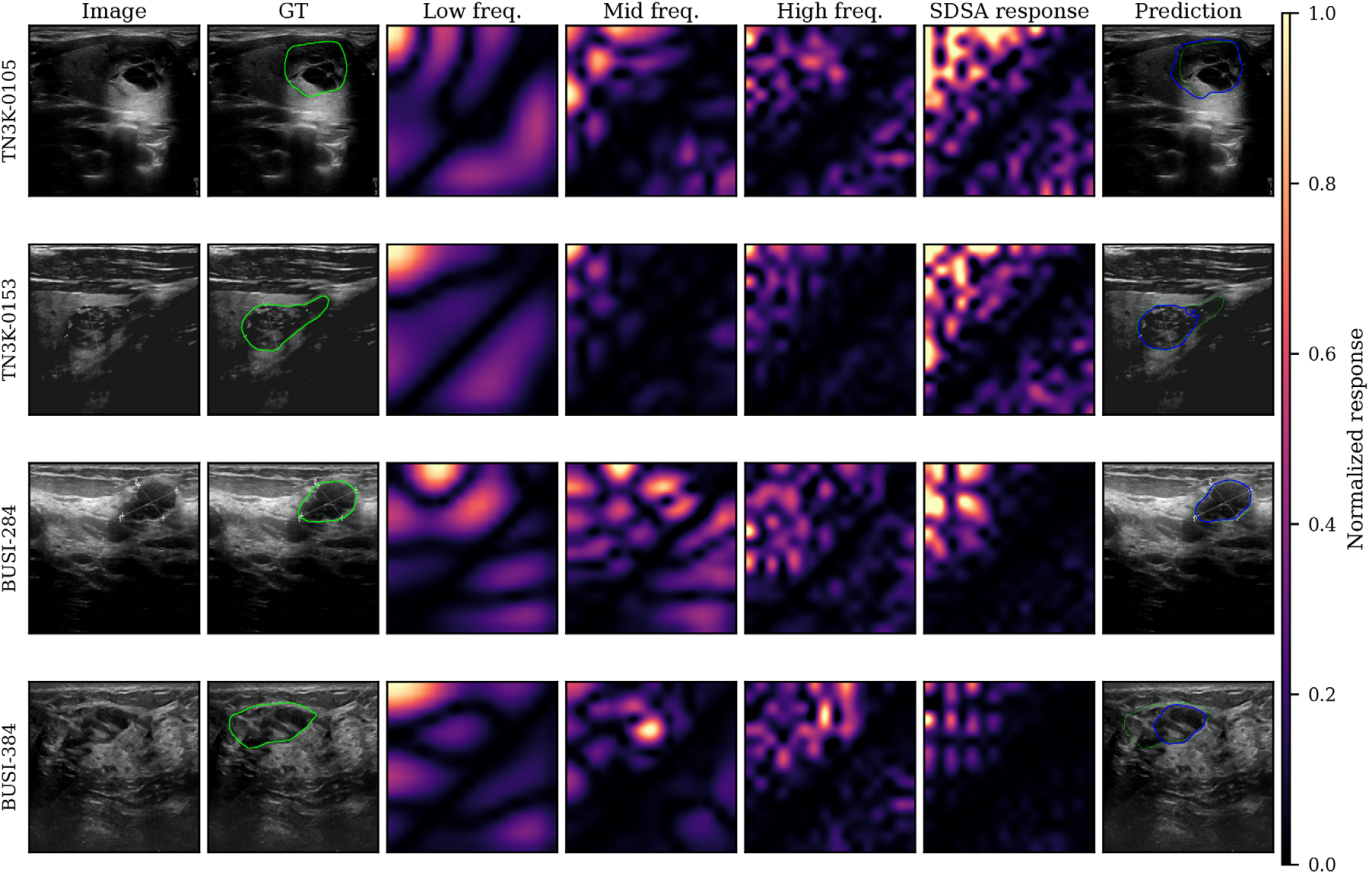
Frequency‐band visualization of representative hard cases. In the displayed samples, low‐frequency responses are spatially broad, mid‐frequency responses are more prominent near some lesion boundaries and local transitions, and high‐frequency responses contain finer and more scattered patterns. The displayed SDSA response is more prominent near some boundary‐related regions; this is a descriptive observation rather than evidence of selective or causal frequency use. Green contours denote ground‐truth masks, and blue contours denote predictions.

In the analyzed samples from both datasets, the radial frequency response analysis shown in Figure 10 indicates stronger low‐frequency responses in non‐boundary regions and a relative shift toward the mid‐to‐high frequency range in boundary regions. Together, the visual and radial‐frequency analyses provide descriptive evidence that the spectral branch produces responses associated with lesion‐boundary regions and may help interpret the ablation results. However, they do not establish causal use or a unique role of these cues, nor do they demonstrate that frequency‐domain information is generally more useful for ultrasound than for other imaging modalities.

**FIGURE 10:**
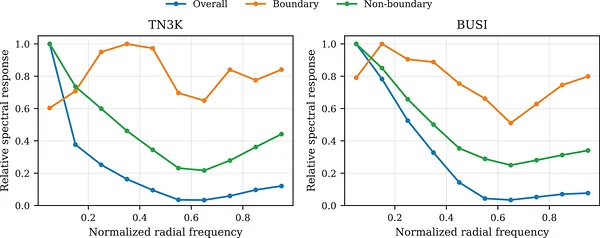
Radial frequency response analysis on TN3K and the BUSI benign subset. In the analyzed samples, non‐boundary regions have stronger low‐frequency responses, whereas boundary regions show relatively stronger responses in the mid‐to‐high frequency range. This pattern is consistent with an association between mid‐to‐high‐frequency responses and boundary regions. Responses are normalized for visualization. The label “BUSI” within the figure denotes the BUSI benign subset.

### 7.3 Justification of channel‐mean spectral tokenization

Channel averaging may reduce channel‐specific spectral variation; therefore, additional channel‐preserving spectral tokenization strategies were evaluated. The channel‐mean DCT branch is not intended to replace the original spatial representation. In SF‐EchoNet, the original spatial feature stream is retained, so channel‐resolved spatial features remain available during subsequent attention computation. The DCT branch provides an auxiliary frequency‐guidance signal derived from channel‐averaged features.

To evaluate whether preserving more channel‐specific spectral information improves performance, the proposed channel‐mean DCT was compared with three alternative spectral tokenization strategies: per‐channel DCT, grouped DCT, and learnable spectral projection. As shown in Table 5, channel‐mean DCT obtained the numerically highest mean mIoU among the four tested strategies on the BUSI benign subset. No inferential statistical comparison among these variants was performed; therefore, the observed differences should be interpreted descriptively and do not identify the mechanism responsible for them.

**TABLE 5:** Ablation study of different spectral tokenization strategies on the BUSI benign subset.

Spectral tokenization strategy	mIoU (%)
Channel‐mean DCT (proposed)	**79.58** ± **0.21**
Per‐channel DCT	79.32 ± 0.17
Grouped DCT (G=8)	78.63 ± 0.25
Learnable spectral projection	78.37 ± 0.19

*Note*: Values are reported as mean ± standard deviation across five runs.

Bold values indicate the best performance in each column.

We further analyzed the similarity of spectral‐energy profiles across feature channels. As shown in Figure 11, the channel‐wise spectral‐energy profiles were highly correlated on both TN3K and the BUSI benign subset, with mean off‐diagonal correlations of 0.960 and 0.957, respectively. Moreover, the cosine similarity between the mean‐DCT spectrum and the channel‐averaged DCT spectrum reached 0.992 on TN3K and 0.964 on the BUSI benign subset. These findings show substantial similarity among the measured channel‐wise spectral‐energy profiles and are consistent with using channel‐mean DCT as a compact spectral representation.

**FIGURE 11:**
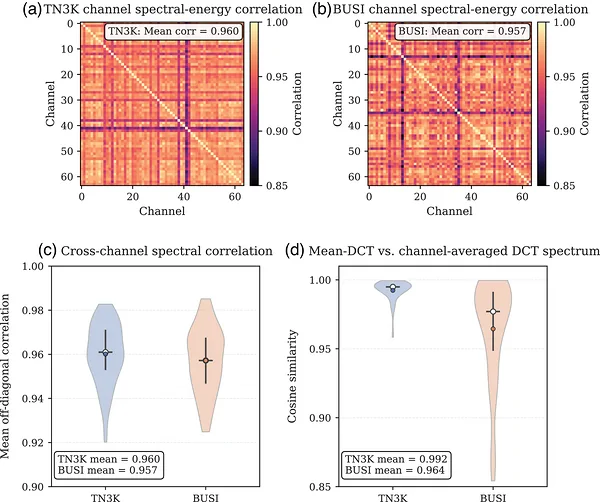
Channel‐wise spectral‐profile similarity analysis. (a,b) Averaged channel‐channel correlation matrices of spectral‐energy profiles on TN3K and the BUSI benign subset. (c) Distribution of mean off‐diagonal channel correlations. (d) Cosine similarity between the mean‐DCT spectrum and the channel‐averaged DCT spectrum. The measured similarities are consistent with using channel‐mean DCT as a compact spectral representation. The color scale in (a,b) is clipped to [0.85, 1.00] for visualization. The label “BUSI” within the figure denotes the BUSI benign subset.

Qualitative visualizations in Figure 12 show spatial spectral‐response patterns for channel‐mean DCT and the per‐channel, grouped, and learnable variants. In the displayed samples, the channel‐mean responses are spatially similar to those of the more complex variants.

**FIGURE 12:**
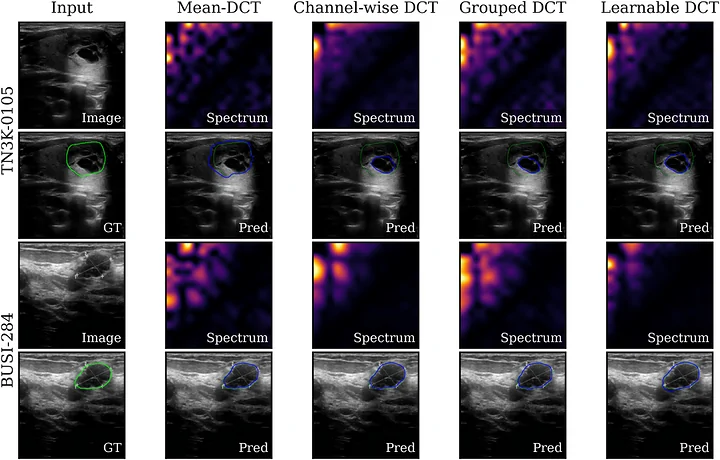
Qualitative comparison of spectral tokenization strategies. The displayed channel‐mean DCT response maps have spatial patterns comparable to those of the per‐channel DCT, grouped DCT, and learnable spectral projection variants. Green contours denote ground‐truth masks, and blue contours denote predictions from the corresponding variants. The spectral response maps are normalized to [0, 1].

Overall, channel‐mean DCT obtained the numerically highest mean mIoU among the four tested tokenization strategies. Because inferential comparisons and variant‐specific runtime and memory measurements were not reported, these results describe its segmentation performance within the tested setting but do not independently establish superiority or an accuracy–efficiency advantage over the alternative tokenization strategies.

### 7.4 Positioning relative to frequency‐domain segmentation methods

The cited frequency‐domain segmentation methods^20^, ^21^, ^22^ use spectral representations to complement spatial‐domain CNN or Transformer features through auxiliary frequency branches, frequency filtering, feature decomposition, or spatial‐frequency fusion. In these cited architectures, frequency information is generally incorporated as an additional representation rather than directly within the self‐attention computation.

SF‐EchoNet differs from these methods in three aspects. First, instead of treating frequency information as a post‐hoc feature enhancement, the proposed SDSA module injects DCT‐derived spectral tokens directly into the self‐attention process; frequency‐domain cues therefore enter the attention computation and modulate spatial correlations during feature interaction. Second, the proposed channel‐mean DCT design provides a compact global spectral representation while preserving the original spatial feature stream for channel‐specific semantic representation. Third, SF‐EchoNet combines spectral‐guided attention with SAFM so that frequency‐enhanced context and encoder features are fused during decoding.

The architectural contribution of SF‐EchoNet is not simply the use of DCT, but the integration of spectral priors into the attention mechanism and decoder fusion process, evaluated here for ultrasound lesion segmentation. The frequency‐band analyses and spectral‐tokenization ablations are consistent with the intended use of the frequency branch as an auxiliary boundary‐related guidance signal, but they do not establish this as its exclusive or causal mechanism.

### 7.5 Ablation analysis of the SAFM

To evaluate the proposed SAFM, we compared it with “Skip fusion (addition)” and “Skip fusion (concat.).” In these baseline variants, the encoder skip features and decoder features are directly combined using static fusion operations. As summarized in Table 6, the five‐run summaries for the SAFM variant were numerically higher for the overlap and pixel‐classification metrics and lower for HD95 than those of the two tested static‐fusion variants on both datasets. These results are consistent with a performance contribution of the complete SAFM under the tested configurations. However, no inferential comparison among these variants was performed, and the ablation does not isolate the individual effects of the channel‐ and spatial‐attention pathways.

**TABLE 6:** Ablation comparison of the SAFM with two static skip‐fusion strategies.

Dataset	Method	mIoU↑	DSC↑	Acc↑	Spe↑	Sen↑	HD95↓
TN3K	**SAFM (proposed)**	**88.01** ± **0.25**	**93.38** ± **0.22**	**97.25** ± **0.14**	**98.79** ± **0.18**	**86.08** ± **0.33**	**41.31** ± **0.97**
	Skip fusion (concat.)	86.53 ±0.23	92.47 ±0.18	97.01 ±0.09	98.31 ±0.09	85.21 ±0.32	44.25 ±0.98
	Skip fusion (addition)	86.02 ±0.26	92.08 ±0.20	96.84 ±0.10	97.89 ±0.10	84.76 ±0.35	45.03 ±1.04
BUSI‐benign	**SAFM (proposed)**	**79.58** ± **0.21**	**87.62** ± **0.18**	**96.75** ± **0.09**	**98.36** ± **0.07**	**75.88** ± **0.45**	**47.22** ± **0.82**
	Skip fusion (concat.)	78.09 ±0.24	86.54 ±0.20	96.39 ±0.10	97.84 ±0.09	74.98 ±0.47	49.15 ±1.00
	Skip fusion (addition)	77.63 ±0.27	86.13 ±0.23	96.12 ±0.11	97.52 ±0.10	74.36 ±0.49	50.02 ±1.08

*Note*: Bold denotes the best result among the tested configurations.

### 7.6 Computational efficiency analysis

To further examine whether the proposed components introduce excessive computational overhead, we conducted a stepwise efficiency ablation study, as summarized in Table 7. Starting from a convolutional decoder baseline, the spatial‐attention path of SDSA increased latency by 1.79 ms, while its DCT spectral‐guidance path introduced the largest additional latency of 3.17 ms. This observation is consistent with the fact that frequency‐domain operations are not always fully reflected by FLOPs estimation but can affect practical runtime. The MR‐FFN block introduced 0.27 ms additional latency; the skip‐feature fusion and channel–spatial attention stages of SAFM added 1.16 and 0.16 ms, respectively. The full SF‐EchoNet model achieved 9.98 ms latency, corresponding to a model‐only throughput of 100.2 FPS under the reported single‐GPU benchmark. End‐to‐end real‐time performance in a clinical acquisition and processing pipeline was not evaluated.

**TABLE 7:** Stepwise computational efficiency ablation of SF‐EchoNet.

Stage	Params (M)	FLOPs (G)	Latency (ms)	FPS	GPU Mem (MB)	Step ΔFLOPs (G)	Step ΔLatency (ms)
Baseline (convolutional decoder)	11.64	12.10	3.51	285.2	111.5	—	—
+ SDSA: spatial‐attention path	11.83	12.45	5.30	188.8	115.2	+0.34	+1.79
+ SDSA: DCT spectral‐guidance path	11.83	12.45	8.47	118.1	140.2	∼0^a^	+3.17
+ MR‐FFN block	12.01	12.77	8.74	114.4	140.9	+0.33	+0.27
+ SAFM: skip‐feature fusion	12.27	13.06	9.90	101.0	141.9	+0.29	+1.16
+ SAFM: channel–spatial attention	12.27	13.07	10.06	99.4	141.9	+0.01	+0.16
**Full SF‐EchoNet**	**12.24**	**12.54**	**9.98**	**100.2**	**141.5**	—	**+6.47 vs. baseline**

*Note*: All measurements were obtained using 512×512 input images with batch size 1 on an NVIDIA RTX A6000 GPU. The latency increment is calculated relative to the previous stage.

a LOPs estimated by fvcore may not fully count FFT/DCT‐related operators; therefore, measured latency and FPS are more reliable for assessing the practical cost of the SDSA DCT spectral‐guidance path.

Bold values indicate the best performance in each column.

The module‐wise results show that the additional computational cost mainly comes from the SDSA DCT spectral‐guidance and spatial‐attention paths, whereas the MR‐FFN block and SAFM channel–spatial attention add less overhead. Under the reported hardware and input configuration, the full model used 12.24M parameters, 12.54G profiled FLOPs, and 141.5 MB peak GPU memory, with a measured latency of 9.98 ms.

### 7.7 Failure‐case analysis and practical considerations

Figure 13 presents four representative cases in which SF‐EchoNet did not produce satisfactory segmentation. In TN3K‐0037, the lesion–background contrast is extremely low and the boundary is visually indistinct, resulting in boundary displacement. In TN3K‐0186, only part of a small lesion is identified. The limited pixel extent of such lesions and feature downsampling may reduce the preservation of discriminative details. In BUSI‐benign‐131, weak contrast and heterogeneous internal echoes are accompanied by an incomplete predicted contour. In BUSI‐benign‐397, the prediction extends substantially into an adjacent hypoechoic region whose appearance is similar to that of the target lesion. These examples suggest that spectral guidance does not fully resolve ambiguity when lesion boundaries are weak, lesions occupy very few pixels, or neighboring tissues have similar echogenicity. Because these observations are based on representative examples rather than a controlled causal analysis, the proposed explanations should be interpreted as plausible contributing factors.

**FIGURE 13:**
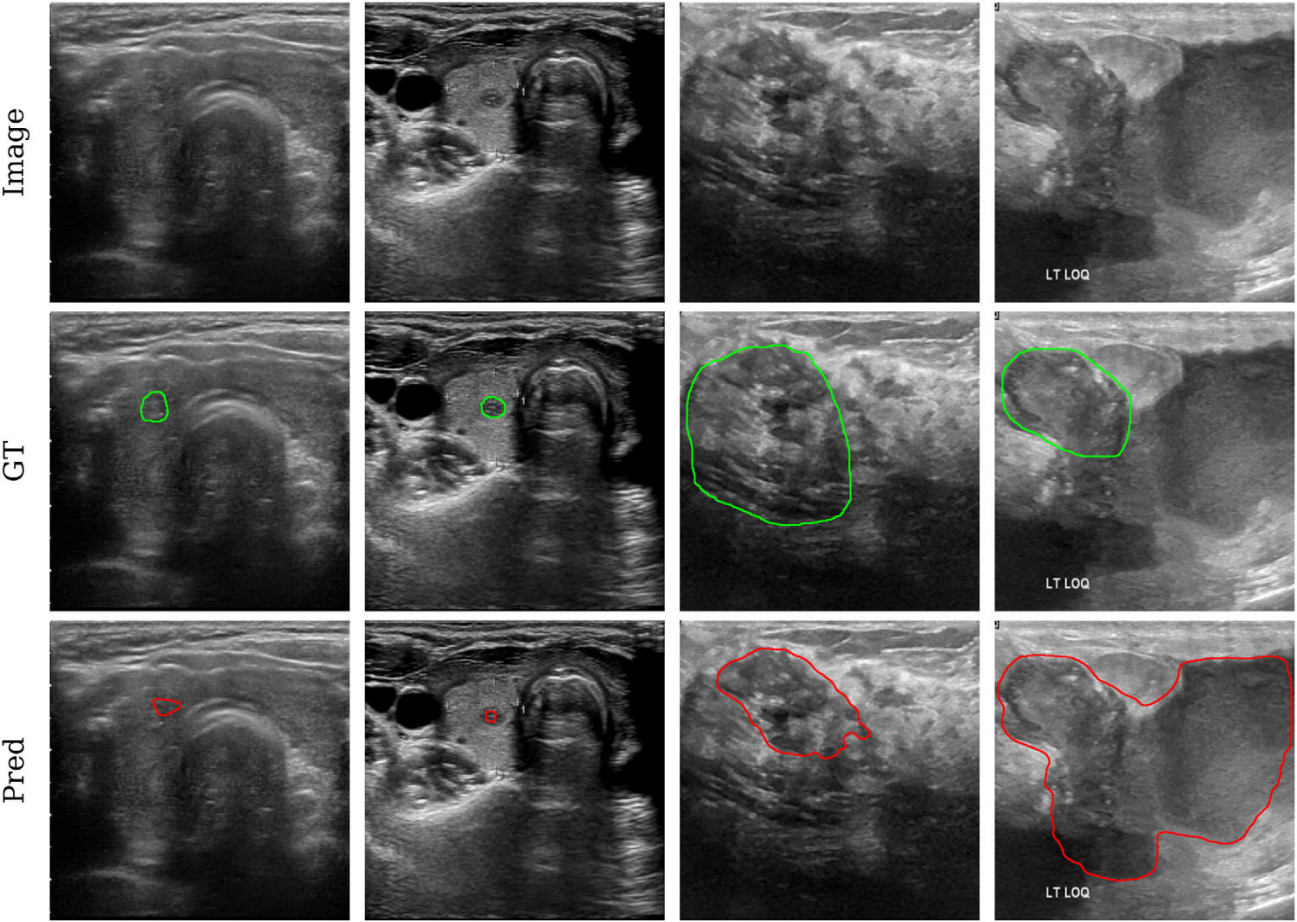
Representative failure cases of SF‐EchoNet. From left to right: TN3K‐0037, an extremely low‐contrast lesion with an indistinct boundary; TN3K‐0186, under‐segmentation of a small lesion; BUSI‐benign‐131, incomplete lesion delineation under low contrast; and BUSI‐benign‐397, severe over‐segmentation into an adjacent hypoechoic region. Green contours denote ground‐truth masks, and red contours denote model predictions.

Users should therefore exercise particular caution when applying SF‐EchoNet to very small lesions, poorly contrasted or partially obscured boundaries, heterogeneous lesions, and images containing adjacent hypoechoic structures. Predicted masks in these settings should be visually reviewed and, when used for quantitative measurements or downstream analysis, corrected by an experienced operator if necessary. The model output should be treated as segmentation assistance rather than an autonomous clinical decision. Future work will investigate small‐lesion‐aware objectives, uncertainty estimation, hard‐case enrichment, boundary‐focused supervision, and multi‐center training to reduce these failure modes and identify cases that require manual review.

### 7.8 Limitations and future work

Although SF‐EchoNet obtained higher overlap metrics than the compared methods on the two evaluated datasets, several limitations remain. First, the failure‐case analysis showed that the model can produce boundary displacement, under‐segmentation, incomplete contours, or substantial over‐segmentation in images with extremely low lesion–background contrast, very small lesions, heterogeneous echoes, or adjacent hypoechoic regions. These errors may affect lesion measurements and downstream analyses; consequently, challenging predictions require visual review and should not be used as autonomous clinical outputs. Second, the model was evaluated only on two‐dimensional B‐mode ultrasound images of thyroid nodules and benign breast lesions. Its robustness across anatomical sites, scanner vendors, transducer frequencies, and acquisition protocols has not been established. Because different imaging settings can produce different spatial‐frequency profiles and artifact patterns, future work should include multi‐center, multi‐device validation and domain‐adaptive training. Extending the framework to three‐dimensional ultrasound and elastography may also help evaluate whether the proposed spatial‐frequency design generalizes to other ultrasound modalities.

Paired image‐level analyses against BDFormer supported differences in mIoU and mDSC on both datasets and in HD95 on TN3K. For HD95 on the BUSI benign subset, the corrected Wilcoxon test was significant, but the bootstrap confidence interval of the mean paired difference crossed zero; therefore, the magnitude of this boundary‐distance difference remains uncertain. Independent cross‐institutional, cross‐device, and prospective validation were not included. TN3K and the BUSI benign subset are public two‐dimensional ultrasound image datasets and cannot fully represent scanner‐, operator‐, and institution‐level variations encountered in clinical practice. Moreover, the BUSI experiment used a random image‐level train‐test split, so the results should not be generalized to malignant breast lesions or broader clinical breast ultrasound screening settings. Future studies should evaluate SF‐EchoNet on multi‐center cohorts acquired with different ultrasound scanners, probes, and imaging protocols, including both benign and malignant breast lesions.

By incorporating frequency‐domain priors into attention‐guided feature interaction, SF‐EchoNet implements explicit spatial‐frequency modeling for ultrasound segmentation. Several questions remain open. First, the current comparison focuses on representative CNN‐, Transformer‐, and frequency‐enhanced segmentation methods under the TN3K benchmark and the BUSI benign subset; additional comparisons with a broader range of frequency‐domain medical segmentation models^20^, ^21^, ^22^ under unified training protocols would further clarify the method's relative position. Second, the analyses identified an association between mid‐to‐high‐frequency responses and boundary regions in the evaluated samples; the optimal frequency decomposition strategy may vary across organs, scanners, and ultrasound acquisition protocols. Third, extending the framework to multi‐center ultrasound datasets, malignant breast lesions, 3D ultrasound, and other sonographic tasks would further characterize the generalizability of frequency‐domain priors.

#### 7.8.1 Downstream clinical utility

This study focuses on lesion boundary delineation in two‐dimensional public ultrasound segmentation benchmarks. It does not directly evaluate pathological classification, radiomics robustness, observer variability, clinical endpoints, or treatment‐planning outcomes. The effect of improved segmentation fidelity on downstream tasks should be assessed in dedicated studies, such as radiotherapy‐specific ultrasound datasets, inter‐ and intra‐observer comparisons, radiomics feature stability analysis, benign‐malignant classification experiments, and prospective clinical evaluation. Such evidence is required before the clinical utility of SF‐EchoNet can be established beyond segmentation benchmarks.

## 8 CONCLUSION

In this work, we introduced SF‐EchoNet, a spatial‐frequency interactive framework for two‐dimensional ultrasound lesion segmentation. Its ESI‐Former block incorporates DCT‐derived spectral representations into self‐attention, while SAFM adaptively integrates encoder skip features with decoder representations during reconstruction. On TN3K and the BUSI benign subset, SF‐EchoNet achieved mIoU values of 88.01% and 79.58% and DSC values of 93.38% and 87.62%, respectively, while obtaining numerically lower run‐level mean HD95 values than the compared methods. Paired image‐level analyses supported the overlap improvements on both datasets, although the magnitude of the mean HD95 difference on the BUSI benign subset remained uncertain. With 12.24M parameters and a latency of 9.98 ms (approximately 100 FPS) under the reported RTX A6000 setup, SF‐EchoNet combined segmentation performance with a relatively small computational footprint. Nevertheless, broader multi‐center and multi‐device validation is required, and predictions for very small, poorly contrasted, or structurally ambiguous lesions should undergo expert review before quantitative or downstream clinical use.

## AUTHOR CONTRIBUTIONS

**Yi Xin**: Conceptualization; methodology; formal analysis; investigation; data curation; writing –original draft preparation. **Shiyou Wei**: Conceptualization; methodology; writing – review and editing; visualization. **Qianping He**: Conceptualization; methodology; writing – review and editing; visualization. **Qingqing Ling**: Software; formal analysis; resources; writing review. **Wei Zhu**: Software; formal analysis; resources; writing review. **Wei Jiao**: Conceptualization; supervision; software; data curation. **Dandan Ling**: Conceptualization; supervision; writing – review and editing. All authors have read and agreed to the published version of the manuscript.

## CONFLICT OF INTEREST STATEMENT

The authors declare no conflicts of interest.

## ETHICAL APPROVAL

This study used publicly available de‐identified ultrasound image datasets and did not involve new human participant recruitment or prospective data collection; therefore, additional institutional ethical approval was not required.

## GENERATIVE AI STATEMENT

During the preparation of this manuscript, the authors used ChatGPT (OpenAI) solely for English language polishing and grammatical correction. All suggestions were reviewed and revised by the authors, who take full responsibility for the final content.

## Data Availability

The authors will share data upon request to the corresponding author.
